# Individual ball possession in soccer

**DOI:** 10.1371/journal.pone.0179953

**Published:** 2017-07-10

**Authors:** Daniel Link, Martin Hoernig

**Affiliations:** 1 Department of Exercise Science and Sport Informatics, Technical University of Munich, Munich. Germany; 2 Department of Computer Science, Technical University of Munich, Munich, Germany; Norwegian University of Science and Technology, NORWAY

## Abstract

This paper describes models for detecting individual and team ball possession in soccer based on position data. The types of ball possession are classified as Individual Ball Possession (IBC), Individual Ball Action (IBA), Individual Ball Control (IBC), Team Ball Possession (TBP), Team Ball Control (TBC) und Team Playmaking (TPM) according to different starting points and endpoints and the type of ball control involved. The machine learning approach used is able to determine how long the ball spends in the sphere of influence of a player based on the distance between the players and the ball together with their direction of motion, speed and the acceleration of the ball. The degree of ball control exhibited during this phase is classified based on the spatio-temporal configuration of the player controlling the ball, the ball itself and opposing players using a Bayesian network. The evaluation and application of this approach uses data from 60 matches in the German Bundesliga season of 2013/14, including 69,667 IBA intervals. The identification rate was F = .88 for IBA and F = .83 for IBP, and the classification rate for IBC was **κ =** .67. Match analysis showed the following mean values per match: TBP 56:04 ± 5:12 min, TPM 50:01 ± 7:05 min and TBC 17:49 ± 8:13 min. There were 836 ± 424 IBC intervals per match and their number was significantly reduced by -5.1% from the 1^st^ to 2^nd^ half. The analysis of ball possession at the player level indicates shortest accumulated IBC times for the central forwards (0:49 ± 0:43 min) and the longest for goalkeepers (1:38 ± 0:58 min), central defenders (1:38 ± 1:09 min) and central midfielders (1:27 ± 1:08 min). The results could improve performance analysis in soccer, help to detect match events automatically, and allow discernment of higher value tactical structures, which is based on individual ball possession.

## 1 Introduction

Technological innovations of recent years, particularly in the field of tracking systems, are leading to an increasing volume of data in sports. These enormous amounts of information present new challenges when it comes to analyzing and interpreting this data. What is required are answers to such questions as how sports clubs can best exploit the possibilities on offer to analyze game tactics, manage training processes and make better transfer decisions; how media companies can use this information to offer better and more innovative match coverage products; and how new scientific insights into the nature of sporting phenomena in general and the factors that influence performance can be gained.

In soccer, *Competition Information Provider*s (CIPs) and mainstream of sports science traditionally use relatively simple performance indicators, such as shots on goal, number of passes, tackles won, team ball possession, distance covered or heat maps [1–3]. While some of these standard indicators can be easily generated from the raw data, their usefulness for performance analysis should be regarded with a certain degree of skepticism [4,5].

In recent years, there has been increased activity in developing more intelligent performance indicators on the part of both the CIPs and the scientific community (an overview is given by [6]). One approach is to model interactions between teams or players based on concepts of dynamic systems theory such as Approximate Entropy or Relative Phase [7–9]. Another group of studies use networks approaches to describe passing behavior and to find patterns which are correlated with success [10–12]. In addition, Machine Learning is also used to study team tactics. Grunz, Memmert and Perl [13] for example use self-organizing maps to classify the behavior of small groups of players in set play situations, such as a game opening sequence. Le et al. [14] use deep learning algorithms to imitate tactical behavior and estimate how each team might have approached a given situation.

**Fig 1:**
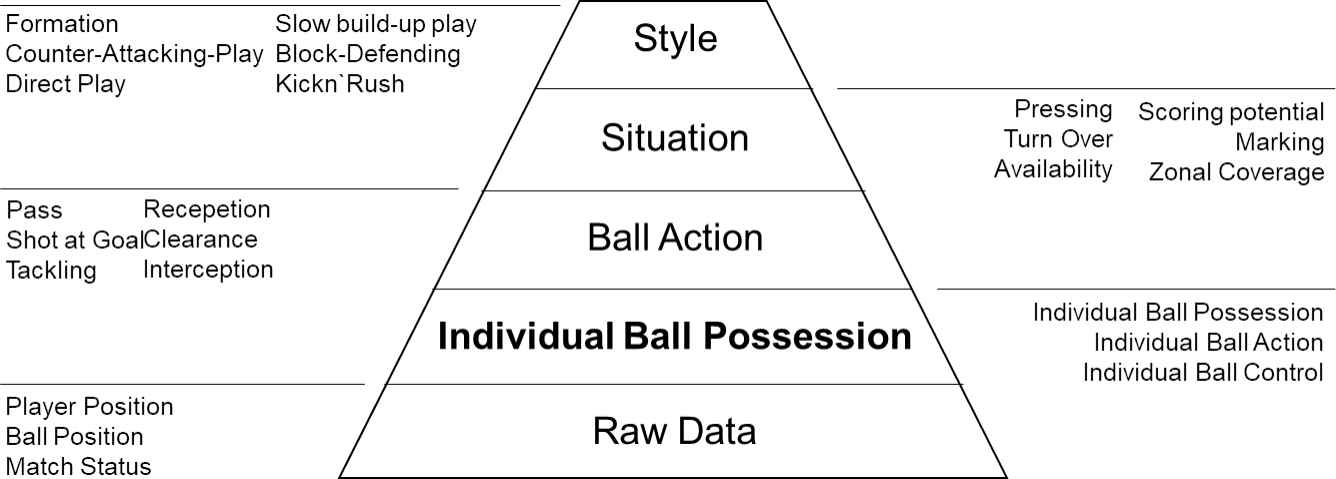
Hierarchy of soccer constructs. Individual Ball Possession and its subtypes are a fundamental prerequisite for being able to discern ball actions. These actions are again the base for identifying situations. The typical behavior in these situations can be aggregated to playing styles.

Another group of emerging approaches to analysis derives from well-known tactical soccer constructs like Control of Space, Availability, Pressing or Dangerousity from spatiotemporal tracking data [15–19]. These construct can be organized using a hierarchy consisting of actions and different types of situations [20], which can be aggregated to playing styles as shown in Fig 1. A fundamental prerequisite when discerning such constructs is to know, which player has the ball. This information is typically not included in the raw data provided by CIPs. The reason is, that ball possession data is collected by human data loggers concurrent with the game in real time, and it would be too expensive to manually record data on an individual player basis. To our knowledge, there are no studies, which deal with the problem of identifying ball possession on an individual level.

Furthermore, Individual Ball Possession is not only important as an auxiliary concept. In soccer`s performance analysis, team ball possession is the most commonly investigated performance indicator [4]. Its relevance is easy to understand, since having control of the ball is a fundamental prerequisite for being able to invade the opposing team's third of the pitch and score goals [21]. As a consequence, successful teams not only have a greater share of ball possession [22,23] but the periods of ball possession are longer too [24]. On the other hand, having more ball possession is not of itself a criterion for success. As such, teams tend to exhibit less ball possession in won games than they do in lost games—a phenomenon that may be explained by a change in tactics depending on if they are in the lead or behind [24,25]. Ball possession is not so much a causal variable but rather the consequence of a process of interaction that is determined by a number of contextual factors, such as the venue, the quality of the opponent, the tactical configuration and the current score [26–29].

This paper is the first to describe how ball possession data can be collected and evaluated not only at the team level, but also at an individual level. There is one paper [30] that determines ball possession using an approach similar to that presented in this paper, but the methods differ in their basic objective, however, as they examine more general aspects of player trajectories using simulated data. Another paper by Carling [31] describes of running activity with the ball, which is similar to our understanding of individual ball possession, but does not consider tactical aspects.

In the following sections, we first define various types of ball possession independent of operational considerations. Computational models are developed for calculating these variants and validated using manually collected reference data. Lastly, we present and discuss some applications for the presented models. The results can be used by other scientific workgroups who need information on Individual Ball Possession to analyze tactics, and by coaches and analysts to improve their capabilities in performance analysis.

## 2 Ball possession types

**Fig 2:**
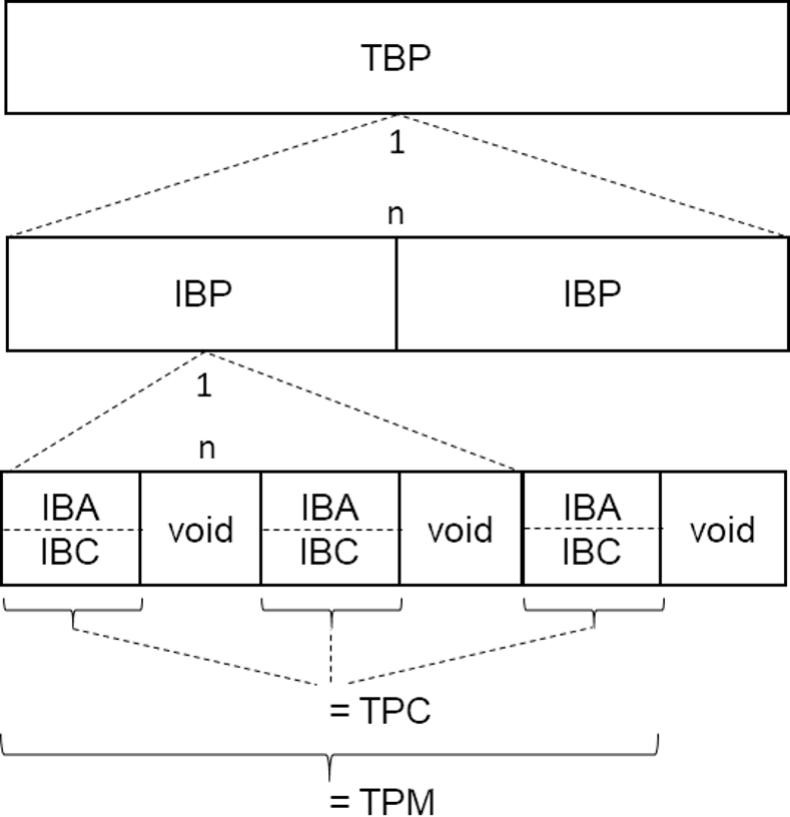
Relationships between the different types of ball possession. Team ball possession (TBP) consists of an unbroken sequence of individual ball possession (IBP) phases. IBP is composed of one or more phases in which an action can be performed with the ball. These are designated individual ball actions (IBAs). It is possible for IBAs to be separated by void phases during which no ball control exists (e.g. while the ball is in the air). An IBC is an IBA where ball control is present. Team ball control (TBC) is the union of all TBC phases. Team play making (TPM) corresponds to TBC plus the intermediate void phases.

In the following, a system of terms is introduced that defines the phenomenon of ball possession and differentiates its various types (Fig 2). In our approach, only time intervals during which the ball is in play are considered when determining ball possession. When the ball is in play, one of the two teams always has team ball possession and one of the players always has individual ball possession. When individual ball possession switches between two players, it is assumed that the pass reflects the tactical intent of the first player. This time interval is thus classified as ball possession for the first player. No individual player and neither team is assigned ball possession when the ball is not in play. This approach differs from that taken by some CIPs, who ascribe these time intervals to one or other of the teams (either the team that had possession of the ball up to that moment or the team that is in possession of the ball thereafter).

One detail of the definition concerns the question of when ball possession begins. In order to simplify the data collection process, CIPs often set this time to the moment of first contact with the ball. In the following, however, the ball is considered to be in a player's possession once it enters that player's action space (for example, a slow ball that a player is close to even though he has not yet made contact with it). Ball possession is thus defined as follows:

1 *Individual Ball Possession (IBP)* begins the moment a player is able to perform an action with the ball (following an IBP of another player or a game interruption). It ends the moment IBP for another player begins.
2 *Team Ball Possession (TBP)* begins the moment IBP for one of the team's players begins (following IBP of a player on the opposing team or a game interruption). It ends with the first IBP for one of the opposing team's players following this.

The time interval in which a player can perform an action with the ball is then separated out from this simple definition of ball possession. This interval no longer includes the time from the ball being passed on until the start of IBP of the next player. It is important to make this distinction in view of the fact that the configuration on the pitch during this time interval determines the tactical options available and their chances of success. The time interval during which no influence can be exerted on the ball is unimportant here.

3 *Individual Ball Action (IBA)* of a player begins the moment this player is able to perform an action with the ball and had no IBA prior to this. It ends the moment the player is unable to perform any further action with the ball.

A further internal distinction within the IBA can be made based on the level of ball control exhibited. What matters here is whether a player has the ball sufficiently under control that he can consciously chose between several play options. An example where this is not the case is when a player attempts to deliver the ball to a specific area of the pitch under extreme pressure (or "ping-pong" sequences in the midfield or the ball being headed on following a cross). This distinction is made because it is only possible to draw reliable conclusions about team or individual tactics during game analysis if the ball is fully under control.

4 *Individual Ball Control (IBC)* for a player begins when IBA for this player begins and he is able to decide between several play options during the IBA. It ends the moment this particular IBA of the player ends.

Two further constructs are defined at the team level. Team ball control is the union of all IBCs, i.e. the period of time during which any player in the team has control of the ball. This very narrow understanding of team ball control is supplemented by the "Team Playmaking" construct. This includes the periods of time during which the ball is being passed between the players of a particular team as well. It is equivalent to the common understanding of team ball control prevalent for coaches today.

5 *Team Ball Control (TBC)* for a team begins when IBC for one of this team's players begins and ends as soon as the IBC of this player ends.
6 *Team Playmaking (TPM)* for a team begins when IBC for one of this team's players begins and the team has had no IBC immediately prior to this. It ends with the last IBC before the next *IBA* of a player on the opposing team.

## 3 Detection of ball possession

**Fig 3:**
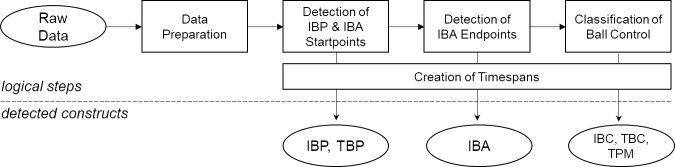
Logical procedural steps for detecting different types of ball possession. Detection is based on identifying the starting and endpoints of IBP and IBA.

Automatic detection of ball possession involves a multi-step process (Fig 3). The first step involves pre-processing raw data provided by the CIPs in order to reduce the number of unrealistic position jumps and noise in the player and ball coordinates. The core of the procedure involves detecting the moments where the IBPs and IBAs start and the IBAs finish. As shown in the following sections, these time codes can be used to reconstruct all of the required time intervals. After that, the level of ball control within the IBAs is estimated using a Bayesian network. The following sections describe the individual steps of the process in detail.

### 3.1 Data preparation

The raw data exists as a sequence of data frames with a frame rate of 25 Hz. This paper only considers the xy-coordinates of the players and the ball, and the running flag (which measures whether the ball is in play versus out of play). Some CIPs also provide z-coordinates as well as a team possession flag, but this information was not used for determining the various types of ball possession that is the focus of this study, because we want to provide a method that works for the raw data of almost all CIPs.

The first step involves identifying unusable sequences in the raw data and excluding them from further processing. First, jumps in the ball's trajectory are detected by applying distance tests on temporally adjacent data points. Very short sections (< 1 s) are excluded from further processing. The Rauch-Tung-Striebel (RTS) method [32] is used to smooth the trajectories of the ball and the players in the remaining sequences. The smoothed data are then used to calculate distances, velocities and accelerations. In the following, for any time code *t*, the position of the *i*th player in the raw data is denoted by $x_{t}^{i}\in R^{2}$ and that of the ball by $b_{t}\in R^{2}$. After applying the stochatical RTS-smoother, the positions are given by ${\underline{x}}_{t}^{i}$ and ${\underline{b}}_{t}$, the speeds by ${\underline{\dot{x}}}_{t}^{i}$ and ${\underline{\dot{b}}}_{t}$ and the acceleration of the ball by ${\underline{\ddot{b}}}_{t}$. The distance *d (a*,*b)* between two positions corresponds to the Euclidean distance.

### 3.2 Detection of IBP & IBA starting point

The IBP or IBA starting point is the moment when a player starts to interact with the ball. We state this as soon as the distance between the player and the ball falls below a threshold value and that player is nearest to the ball. Put formally, this means that both the formula

$$d({\underline{x}}_{t}^{i},{\underline{b}}_{t})<T_{P}$$

**Fig 4:**
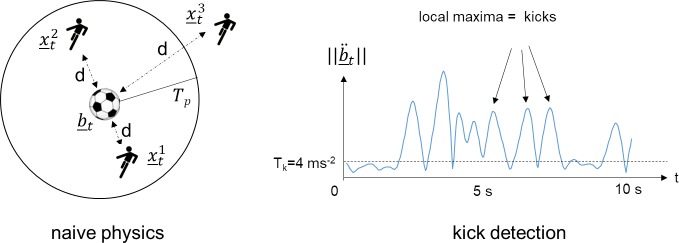
Illustration of IBA starting point determination according to the naive physics model, ball possession exists at time t when the separation d between player(${\underline{x}}_{t}^{i}$) and Ball (${\underline{b}}_{t}$ is less than the threshold *T*_*p*_. Ball possession is attributed to the player closest to the ball (smallest ${\underline{x}}_{t}^{1}$ here). With kick detection (right), the acceleration of the ball ${||\underline{\ddot{b}}}_{t}||$ (smoothed, norm of xy-components) must simultaneously show a local maximum of at least 4 ms^-2^.

Becomes true at time *t* and the distance on the left-hand side of the equation is not smaller for another player id *j* ≠ *i*. The value *T*_*P*_ determines the threshold value for a player being able to physically interact with the ball and has to be trained a priori. We refer to this method as *naive physical* (Fig 4, left).

As stated earlier, the ball position is provided without a z-coordinate. If the ball passes above a player, this could easily lead to possession being incorrectly attributed. We obviate this problem by applying not only a 2d separation threshold, but also by testing to check if a player has interacted with the ball. We use the local maxima ball accelerations together with narrow tolerance to filter out noise. A player only gains ball possession if the requirements for *naive physical* are fulfilled (using another threshold value *T*_*K*_) and a local maxima with a minimum acceleration of 4 ms^-2^ can be detected. We refer to this method as *kick detection* (Fig 4, right).

### 3.3 Detection of IBA endpoint

While the IBP endpoints are known once the IBA starting points have been identified (a player's ball possession ends when there is an interruption of play or when another player has IBP), the IBA endpoints must be specifically identified. There are three possible ways an IBA for a player can end: 1) the game is interrupted, 2) another player gains IBA or 3) the player is no longer able to interact with the ball. Whereas the first two cases are trivial to detect (by checking the running flag or by detecting a new IBA starting point for a different player) the last one requires special treatment. This involves checking whether a player will be still able to interact with the ball within a certain time span. This is true if, for example, a player kicks the ball a few meters ahead of himself while dribbling, but not after making a pass or taking a shot at goal.

**Fig 5:**
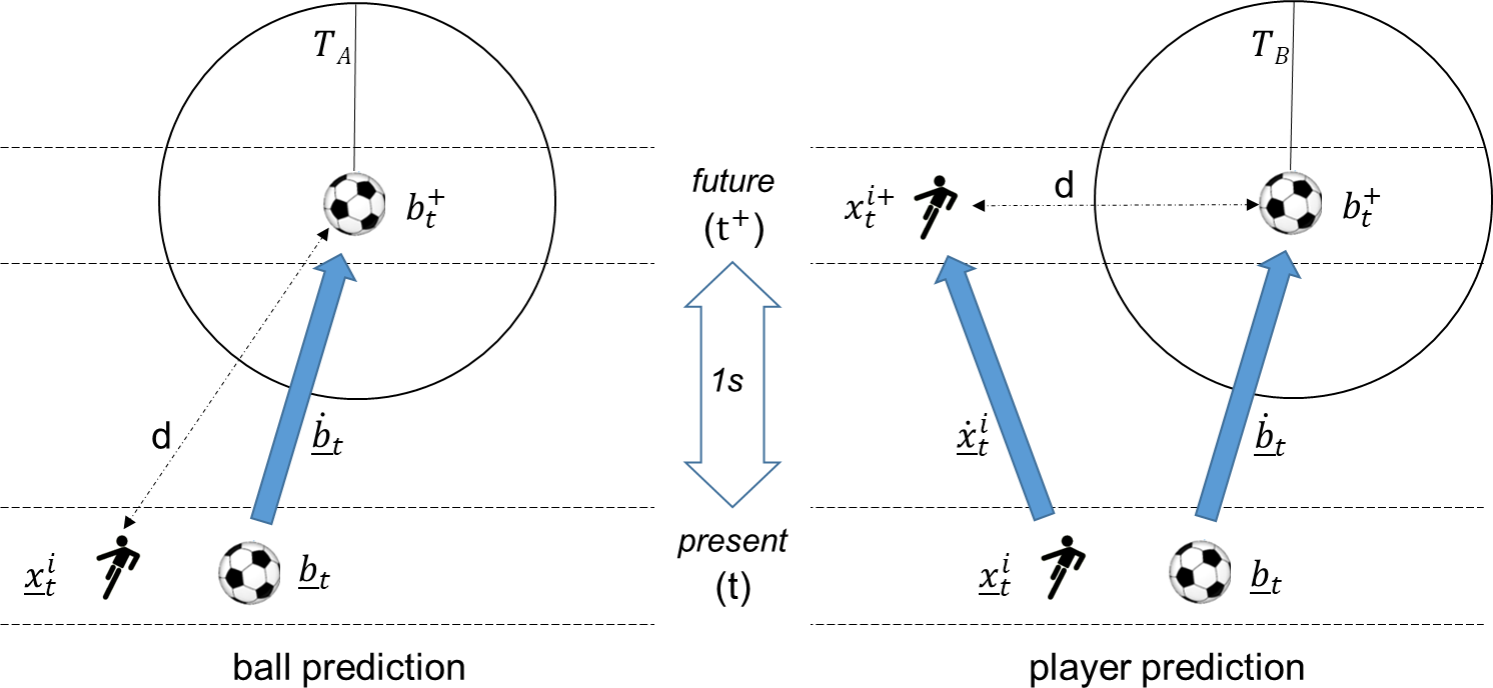
Detection of IBA endpoints: An endpoint exists as soon as the player is unable to interact with the ball for a period of one second. This is the case after a shot on goal or a pass, for example, but not when the player is dribbling the ball stated formally, when using the ball prediction method, the endpoint t of an IBA phase is arrived at when the distance d between ball position ($b_{t}^{+}$) and player position (${\underline{x}}_{t}^{i}$) is no longer below the threshold value (*T*_*A*_) for a period of one second (*t*^+^) at the ball's current speed and direction (${\underline{\dot{b}}}_{t}$). The player's position is assumed to be constant. The player prediction method additionally takes into account the player's future position $x_{t}^{i+}$ based on his direction and speed (${\underline{\dot{x}}}_{t}^{i}$).

Two approaches are presented here. Both make use of the current positions and velocities of the ball or the ball and player to give an estimate of their future locations. Beginning with a constant prediction interval of 1*s*, we can define a future ball position as

$${b_{t}}^{+}≔{\underline{b}}_{t}+{\underline{\dot{b}}}_{t}$$

(to simplify the equations, we omit the underscore for new variables). The individual ball action interval is now defined to end at the first moment t after it began in which the statement

$$d({\underline{x}}_{t}^{i},{b_{t}}^{+})>T_{A}$$

is true. Again, the threshold value *T*_*A*_ has to be trained a priori. In other words, as long as it is possible for the player currently in possession of the ball to control the ball in the near future, he will retain ball possession. This *ball prediction* model (Fig 5, left) can be refined still further by incorporating a prediction about the player's future position

$$x_{t}^{i+}≔{\underline{x}}_{t}^{i}+{\underline{\dot{x}}}_{t}^{i}$$

and changing the inequality to

$$d{(x}_{t}^{i+},{b_{t}}^{+})>T_{B}$$

We refer to this as *player prediction* (see Fig 5, right). In contrast to the previous method, the distance check now incorporates two predictions of future position.

### 3.4 Classification of IBC

Once the IBA starting and endpoints have been detected, the IBA intervals can also be derived. The central question is therefore which of the intervals represents a segment that features ball control. This obviously depends upon a great number of factors that may not be possible to ascertain from positional information alone. For this reason, we decided to use a Bayesian network to classify ball control based on the following features (referring to a particular IBA interval): IBA duration, average ball velocity and acceleration, variance of ball velocity and acceleration, average distance between the ball and the player in possession of the ball and the number of opposing players within certain distances (0.5 m, 1 m, …, 5 m). Training of the net was performed using the K2 algorithm [33].

### 3.5 Creation of time intervals

The classified moments can now be used to unambiguously calculate both the IBPs and the IBAs. Status transitions in ball possession are also determined technically during interruptions in play. Phases when the ball is not in play are not excluded until the time intervals are calculated. This allows for greater tolerance of imprecision when it comes to the running flag.

In this sense, an IBP interval is derived from a determined starting point. The next detected IBA starting point or an interruption in play functions as the endpoint. IBA intervals are calculated from pairs of starting and endpoints (while taking into account interruptions in play). The intervals are each tagged with the player in control of the ball. The team-specific construct TBP is the union of all IBP intervals for a particular team. The TBC is the union of the IBCs of all the players in a team; the TPM, on the other hand, is, according to the model, the result of two new sequences of intervals (one for each team). For each interval in a sequence, the starting point is the beginning of the first IBC of a team following a stoppage or the opposing team being in possession of the ball. The end of such a sequence is the final IBC of the associated TBP phase.

### 3.6 Limitations

The procedure does not consider all special cases that might occur during a match. Firstly, for being able to perform an action with the ball, it is not obligatory to touch it. In the case that a player runs beside the ball for a quite long period without kicking it, the IBA interval would start in the moment of the first touch, which is maybe too late. On the other side, it is possible to touch the ball without reaching the acceleration threshold. This could especially happen, when the contact does not change the ball direction substantially. In other cases, it could be wrong to assign ball possession to the nearest player, e.g. when this player is standing with his back to the ball. Also the assessment of ball control depends on the individual skills of a player and is beyond the scope of this paper.

## 4 Training & evaluation

The training and evaluation phase had two sub-goals: Firstly, training was performed to determine the model parameters. Secondly, the quality of IBP, IBA, IBC detection was examined. An evaluation of the team-specific constructs TBP and TBC was not necessary, since they are derived entirely from the IBPs and IBCs.

### 4.1 Training of Threshold Values & Bayesian Network

Five matches of the sample descripted in Section 5 served as the test sample for training and evaluation. An annotation with reference data (ground truth), which was manually logged by a trained, independent observer post-match, formed the basis for the evaluation. The observer had a panoramic video image of the game at his disposal together with a graphic overlay of positions, frame-by-frame playback control and as much time as needed. A total of 6,976 IBA phases with starting and endpoints were identified. 6,340 of these included ball control.

The training to establish the threshold values *T*_*P*_, *T*_*k*_, *T*_*A*_, and *T*_*B*_ that appear in the model was performed using the reference data. A full grid search was performed in order to determine the global optimum for each of the model's threshold values (Powell, 1988). The Bayesian network described in Section 3.4 was trained using data from the second half of the matches and evaluated using data from the first half. The test data contained 1,674 IBA intervals of which 107 involved no IBC. The aim was to identify these. The training data exhibited a similar ratio with 1,583 to 122.

### 4.2 Quality of IBA & IBP detection

The verification of the running flags and detection of IBA starting and endpoints was performed by comparing them with the respective status change in the ground truth. A change detected in the data is interpreted as a *true positive* (TP) if a change is also present in the ground truth within a 0.6 s interval and—in the case of ball possession—the associated player is correct. A *false negative* (FN) occurs when there is a change in the ground truth that is not detected in the data. A change is classified as a *false positive* (FP) if there is a change detected in the data that is not present in the ground truth.

The metrics PRECISION, RECALL and F-SCORE were used to assess the quality of the recognition system, as is usual in machine learning [34]. A TIMELINE quantity is also introduced. This involves the superimposition of the time axes for the ground truth and the IBP or IBA data with respect to ball possession and comparing each individual point in time. A point in time is adjudicated correct if the same ball possession was present in the ground truth within the specified time window of 0.6 s. The ratio of correct time codes to the number of time codes within the net playing time is recorded as a TIMELINE quantity.

Table 1 shows the identification rates for IBP and IBA. The results suggest that both *kick detection* and *ball prediction* are the most promising models. Although there is greater inaccuracy with regard to ball position, prediction of a player's position and the knowledge of the player's speed do not lead to better concordance as regards classifying ball possession, at least not according to the selected model. In terms of IBA (ball prediction), the data have a 5.9 with respect to timeline.

**Table 1:** Quality of identification of different individual ball possession intervals. IBP^N^ = Starting point: Naive physical, Endpoint: /; IBP^K^ = Starting point: Kick detection, Endpoint: /; IBA^B^ = Starting point: Kick detection, End point: Ball prediction; IBA^B^ = Starting point: Kick detection, End point: Player prediction.

	RECALL	PRECISION	F-SCORE	TIMELINE
IBP^N^	.90	.68	.77	.82
IBP^K^	.80	.86	.83	.87
IBA^B^	.86	.91	.88	.92
IBA^P^	.84	.92	.87	.91

When analyzing video sequences for the FP and FN intervals we found, that many errors can be attributed to tracking losses and running flag inaccuracies. These are fundamental problems that similarly affect every CIP and every game. Some CIPs are already offering to manually edit the tracking data afterwards in order to increase the quality of the data. Nevertheless, methods of analyzing play structures must take these fundamental flaws in the raw data into account and implement appropriate error detection and correction procedures.

On the other hand, one can safely conclude that the quality of tracking has arrived at a level allowing for tactical structures to be reliably detected. The rate of detection of individual ball possession should be good enough for answering many questions regarding performance. As tracking quality will surely increase in the next years due to the technological progress of CIPs, detection will also become more stable. However, current quality clearly exceeds the accuracy of manual acquisition by human game loggers (52%). It is easier for a computer to observe the tolerance of 0.6 s than it is for a human. In addition, manual data acquisition involves a great deal of effort and is only performed at the team level.

### 4.3 Quality of IBC classification

The quality of recognition of IBCs for a given IBA interval was also determined by a comparison with the ground truth. All IBA sequences from the second half (n = 1,674) were used as the data set here. Table 2 shows the results as a confusion matrix.

**Table 2:** Confusion matrix showing the quality of IBC classification.

		Ground Truth	
		IBC	No IBC
**Bayesian Network**	**IBC**	1535	23
	**No IBC**	32	84

97.0% of intervals involving ball control were correctly classified. If no ball control was present, however, only 50% of the intervals could be correctly classified. Overall, 96.7% of the intervals are correctly attributed. The degree of consistency according to Cohen is κ = .67. However, with only 122 non-IBC intervals, the training set for differentiating between IBAs and IBCs is very small. Moreover, the inter-rater reliability test between two human observers on a subset of the intervals (n = 98) did not show complete consistency either (κ = .72). This indicates that it may not be possible to fully objectify the ball control construct. This is quite typical for non-trivial tactical concepts and has also been reported e.g. for the Dangerousity metric [19].

## 5 Game analysis

As a first application of the method, we evaluated new performance metrics based on the different types of individual ball possession. Our sample comprises 60 matches during the 2012/13 seasons of the German Soccer Bundesliga. The positional data were collected during the match using an optical tracking system (TRACAB). Kick detection was used to calculate the IBP and TBP, IBA, IBC and TBC intervals were determined using ball prediction without player prediction. Overall, we collected 69,667 IBA intervals, including 53,354 IBCs.

### 5.1 Team based metrics

Table 3 shows the evaluation of gross game time, net game time (excluding game interruptions), TBP, TMP and TPC. TBP is equal to the net playing time by definition. Since not every TBP involves ball control (e.g. in the case of ping-pong sequences in the midfield) and final phases that do not exhibit control are not counted in this time interval either, the TPM is lower than TBP. The accumulated time of TBC intervals is even lower, causing the time from the ball being passed on until the start of the next player`s IBC to be excluded. The ball possession variables at team level are moderated correlated with net playing time (TPM (min), r = .40; TBC (min), r = .38; TBC (n), r = .24).

**Table 3:** Team based metrics. Playing time, TPB, TPM, TBC according to game section and team status.

		Game Section		Team Status	
	∑	First Half	Second Half	Home	Away
PT gross (min)	93:32 ± 1:36	45:50 ± 0:45	47:43 ± 1:22	28:28 ± 6:06	27:35 ± 6:35
PT net (min)	56:04 ± 5:12	28:22 ± 3:19	27:43 ± 2:38	25:32 ± 5:45	24:39 ± 5:09
TBP (min)	56:04 ± 5:12	14:29 ± 3:23	13:53 ± 3:55	9:14 ± 5:12	8:34 ± 4:34
TPM (min)	50:01 ± 7:05	12:54 ± 2:51	12:27 ± 2:43	429 ± 243	407 ± 222
TBC (min)	17:49 ± 8:13	4:44 ± 2:42	4:25 ± 2:28	1:39 ± 0:56	1:32 ± 0:49
TBC (n)	836 ± 424	208 ± 123	209 ± 116	76 ± 43	73 ± 40
TBC (min^-10 min^)	3:11 ± 1:28	0:51 ± 0:29	0:47 ± 0:26	28:28 ± 6:06	27:35 ± 6:35
TBC (n^-10 min^)	149 ± 77	39 ± 22	37 ± 21	25:32 ± 5:45	24:39 ± 5:09

A paired t-test shows that net game duration was reduced by -2.4% (effect size d = 0.2) from 2^nd^ to 1^st^ half (t = 2.0, p < .05) although gross game duration in 2^nd^ half was +4.1% (d = 1.8) higher compared to 1^st^ half (t = 2.0, p < .001). A similar significant reduction can be observed for TPC(n) and TPC (min). This might be explained by the tactical use of game interruptions in soccer. Siegle and Lames [35] found out that goal kicks or free kicks e.g. goal kicks by the leading team take longer towards the end of the match. Also, there might be a tendency of home teams to have more ball possession, which was also reported by Lago-Peñas and Dellal [25], but the differences do not reach significance level (α = .05) in this sample.

In order to exclude the effect of different net playing time on ball possession variables, we also calculated the number and time of TBC per 10 minutes of net playing time. TPC (n^-10min^) was reduced by -5.1% (d = 0.1) from 1^st^ to 2^nd^ half (t = 2.1p < .001). The same can be observed for TPC (n^-10min^). This finding suggests that the reduction of individual ball possession is not only attributed to less net playing time, but also to a change of game dynamics. This is in line with the findings of Harper et al. [36], who reported a reduced number of passes in the extra time of matches and other studies, that found a decline in running activity from 1^st^ to 2^nd^ half [37,38]. One explanation for this could be fatigue, caused by physiological factors, or a conscious or subconscious pacing strategy [39]. Whatever the case, our data at least suggest that these factors also influence individual ball possession.

### 5.2 Player based metrics

Table 4 shows the values for IBP, IBA and IBC at player level. Data from players, who played less than 45 min in a match, was excluded. In general, one can see that IBP (n) is somewhat smaller than IBA (n), because IBP can include several phases of IBA (if the ball is briefly outside the player's area of action, but subsequently regains control of the ball without any intervening IBA of an opposing player). IBP (n) data corresponds to the "ball contacts" construct that match data providers collect manually using loggers. IBC is lower than IBA, as is also seen at the team level, because not every ball contact involves ball control.

**Table 4:** Player based metrics based on individual ball possession according to playing position. ^-10 min^ indicates occurrences / time per 10 min net playing time. IBC (sn^-1^) represents mean time per interval. Playing positions are GK = Goalkeeper, FB = full back, CD = central defender, WF = wide midfielder, CM = central midfielder, CAM = central attacking midfielder, WI = Winger, CF = central forward and ALL = all positions.

Position	IBP (n^-10 min^)	IBA (n^-10 min^)	IBC (n^-10 min^)	IBC (min)	IBC (min^-10 min^)	IBC (sn^-1^)
GK	9,8 ± 4.3	10,1 ± 4.2	8.8 ± 4.8	1:38 ± 0:58	17.3 ± 10.1	2.1 ± 0.8
FB	14.8 ± 6.8	15.0 ± 6.7	11.7 ± 7.7	1:18 ± 0:56	13.8 ± 10.0	1.2 ± 0.4
CD	15.5 ± 7.8	15.9 ± 7.8	12.8 ± 8.3	1:38 ± 1:09	17.1 ± 12.2	1.3 ± 0.4
WM	13.7 ± 7.2	13.8 ± 7.2	10.2 ± 7.7	1:17 ± 1:08	13.6 ± 12.1	1.2 ± 0.6
CM	16.5 ± 8.6	16.8 ± 8.5	12.8 ± 9.0	1:27 ± 1:08	15.2 ± 12.0	1.2 ± 0.4
CAM	14.0 ± 7.3	14.3 ± 7.5	10.2 ± 8.0	1:17 ± 0:58	13.6 ± 10.2	1.1 ± 0.6
WI	11.9 ± 7.7	12.2 ± 7.8	7.9 ± 7.3	1:04 ± 0:54	11.4 ± 9.7	1.1 ± 0.7
CF	10.1 ± 6.4	10.6 ± 6.4	6.6 ± 6.0	0:49 ± 0:43	8.7 ± 7.6	0.9 ± 0.6
ALL	13.3 ± 7.2	13.6 ± 7.2	10.1 ± 8.1	1:21 ± 1:13	13.8 ± 12.6	1.2 ± 1.0

**Fig 6:**
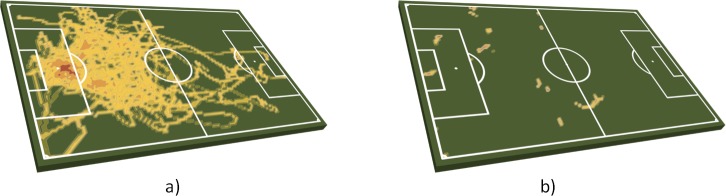
Heatmaps based on all positions (left) compared to positions during IBC (right).

The average IBC duration, i.e. the mean time interval in which a player in possession of the ball can make and execute a tactical decision, was 1:21 ± 1:13 min for players who played for the entire gross match duration. A one-way ANOVA indicated significant differences between the tactical positions for the count of IBP (F = 19.8, p < .01), IBA (F = 19.1, p < .01) and IBC (F = 15.5, p < .01). The highest number of ball possession intervals were found for central midfielders (CM), followed by central defenders (CD). Goalkeepers (GK) and central forwards (CF) showed the lowest numbers of ball possession intervals. This is easy to explain since CM and CD are most responsible for play making. Regarding their relative position in the team formation, CF are the closest to the opponent’s goal which means, that they are most difficult to reach by passing. This is supported by Fig 6, which shows a “traditional” heatmap, including all positions during the match (a) for a CF in contrast to a heatmap based only on the time periods where the player owned IBC (b). Most of the IBC intervals occur in the opponent’s half.

It may also be possible to draw conclusions about the playing characteristics of a playing position by comparing the duration of the ball possession intervals. Although there is a strong correlation between IBC (n) and IBC (min) (r = .85), significant differences in the average lengths of the IBC phases can be detected (F = 53.5, p < .01). For example, CFs have the shortest average ball contact time (0.9 ± 0.6 s), which can be explained by what happens when they had possession of the ball (most of the time being immediately tackled by an opposing player). In contrast, GKs had the longest IBC intervals and the highest proportion of IBA intervals with control. The reasons might be that they are allowed to catch the ball and then are not open to attack.

## 6 Conclusion

The evaluation suggests that the method is suited for determining individual ball possession in soccer. It can be used for a wide variety of potential applications. Firstly, it allows quantification of the amount of time a player in possession of the ball spends in different areas of the pitch or the distribution of ball possession times. Secondly, information about basic events, such as passes, tackles, or shots on goal, can be deduced directly from the individual ball possession data. These events are logged by CIPs as default, but due to the manual data collection process the event lists are not always complete and the events' time stamps are sometimes imprecise. Automatic detection methods can thus help to ensure the quality of match data and potentially reduce the loggers' workload. Thirdly, being able to detect ball possession is a fundamental prerequisite for discerning higher value tactical structures, such as the ability for players to receive a pass, pressing strategies or marking tactics. Finally, the ability to recognize ball possession types holds considerable potential for improving the quality of match analysis in professional soccer.

## Supporting information

S1 File Example of IBA intervals. (ZIP)
